# TLR2 – an innate immune checkpoint in multiple sclerosis

**DOI:** 10.18632/oncotarget.6031

**Published:** 2015-10-08

**Authors:** Md Jakir Hossain, Radu Tanasescu, Bruno Gran

**Affiliations:** Division of Clinical Neuroscience, University of Nottingham School of Medicine, C Floor, South Block, Queen's Medical Centre, Nottingham, United Kingdom

**Keywords:** Toll-like receptor, T cell, multiple sclerosis

Our appreciation of the role of the innate immune system in the organ-specific autoimmunity is growing in parallel with better understanding of innate immunity and its function. Historically, more research had been devoted to adaptive immunity in initiating tissue damage in organ-specific autoimmune diseases, such as multiple sclerosis (MS). MS is an inflammatory demyelinating disease of the central nervous system (CNS) in which immune tolerance to myelin antigens is thought to break down as a consequence of an interaction between genetic predisposition and environmental triggers. A critical role for T- and B-cell receptor mediated adaptive autoimmune responses in MS has been most recently highlighted by genome-wide association and epigenetic fine mapping studies. However, the potential for innate immune receptors to promote autoimmunity is increasingly recognised through effects on antigen presenting cells, including CNS-resident microglia and infiltrating monocytes, but also through direct modulation of adaptive T and B cells.

Toll-like receptors (TLRs) are pattern recognition receptors that play a central role in the initiation of innate immunity against invading pathogens. Initially thought to be exclusively expressed on antigen-presenting cells (APCs: dendritic cells, macrophages, B cells), TLRs are expressed by T-cell populations and play important roles in modulating both T cell effector functions and T cell regulatory (Treg) responses.

Data published over the past 10 years point towards a dual role of TLR2 as both a proinflammatory and anti-inflammatory receptor involved in both peripheral and central innate immune responses. In our recent contributions to the *Journal of Immunology*, we reported that the cell surface innate immune receptor TLR2 modulates the phenotype and function of human CD4^+^CD25^hi^CD127^neg/low^ Tregs and their naïve and memory subsets [1]. TLR2 is preferentially expressed by Tregs and forms heterodimers with either TLR1 or TLR6. We showed that stimulation with Pam3Cys, a TLR1/2 heterodimer agonist, reduces Treg suppressive function and skews them into a T-helper 17 (Th17)-like phenotype in healthy subjects [2]. These findings are supported by previous data in the mouse as well as in human cells, with the interesting exception of HSP60 increasing Treg function through TLR2 [3]. We further explored the relevance of TLR modulation of Treg function in MS [1], and found that TLR2 expression was higher in Tregs from MS patients than in healthy controls. In addition, Tregs from the MS group were more susceptible to TLR2-mediated loss of suppression and Th17 skewing than those from HCs [1]. In MS, functional defects in Treg function are thought to contribute to a failure of immune tolerance, leading to autoimmune attack of myelin antigens in the CNS initiated by autoreactive T cells [1]. In the scenario of an infection, a frequent occurrence in patients with MS, stimulation of TLR2 may therefore enhance pathogen clearance by decreasing Treg-mediated suppression of effector T cells, but potentially at the cost of collateral, autoimmune tissue damage.

The effects of TLR2 stimulation on APCs in the periphery are also important in MS. Correale et al. [4] observed that TLR2 activation on human APC such as dendritic cells and B cells by helminths modulates their cytokine profile towards an anti-inflammatory response. Intestinal commensal bacteria confer protection against CNS demyelination and inflammation during experimental autoimmune encephalomyelitis (EAE), an animal model of MS, through TLR2-mediated CD39 signalling [5].

In the CNS, TLR2 can contribute to neuroinflammation through a PARP1-dependent pathway as showed in a progressive EAE model [6]. By contrast, TLR2 stimulation in the CNS can have neuroprotective roles as well. The accumulation of the small heat shock protein alpha B-crystallin (HSPB5) by stressed oligodendrocytes in brain tissue from people with MS triggers a TLR2-mediated protective response in surrounding microglia. Such protection can be disrupted by IFN-γ produced locally during inflammatory demyelination [7].

What are the implications of the effects of TLR2 modulation in MS and what is the relevance to MS treatments? Firstly, it is known that infections may influence disease susceptibility and the clinical course of MS. During an infection, the activation of TLR1/2 heterodimers by infectious stimuli could exacerbate the known defect in regulatory T-cell function in MS. Secondly; our observation that TLR2 stimulation promotes inflammation in MS would suggest that TLR2 could be a treatment target. Nevertheless, due to its dichotomous role as both pro- and anti-inflammatory molecule, TLR2 modulation is a difficult task. Besides, TLR2 effects are largely tissue- and cell type-dependent. More data on the consequences of TLR2 modulation (such as on the role played by TLR2 during disease exacerbations and remissions) are needed to understand the role of this fundamental regulatory checkpoint in MS.
